# Targeting von Willebrand Factor in Ischaemic Stroke: Focus on Clinical Evidence

**DOI:** 10.1055/s-0038-1648251

**Published:** 2018-05-30

**Authors:** Nina Buchtele, Michael Schwameis, James C. Gilbert, Christian Schörgenhofer, Bernd Jilma

**Affiliations:** 1 Department of Clinical Pharmacology, Medical University of Vienna, Vienna, Austria; 2 Department of Emergency Medicine, Medical University of Vienna, Vienna, Austria; 3 Band Therapeutics, LLC, Boston, Massachusetts, United States

**Keywords:** stroke prevention, von Willebrand factor, high shear rates, individualized treatment

## Abstract

Despite great efforts in stroke research, disability and recurrence rates in ischaemic stroke remain unacceptably high. To address this issue, one potential target for novel therapeutics is the glycoprotein von Willebrand factor (vWF), which increases in thrombogenicity especially under high shear rates as it bridges between vascular sub-endothelial collagen and platelets. The rationale for vWF as a potential target in stroke comes from four bodies of evidence. (1) Animal models which recapitulate the pathogenesis of stroke and validate the concept of targeting vWF for stroke prevention and the use of the vWF cleavage enzyme ADAMTS13 in acute stroke treatment. (2) Extensive epidemiologic data establishing the prognostic role of vWF in the clinical setting showing that high vWF levels are associated with an increased risk of first stroke, stroke recurrence or stroke-associated mortality. As such, vWF levels may be a suitable marker for further risk stratification to potentially fine-tune current risk prediction models which are mainly based on clinical and imaging data. (3) Genetic studies showing an association between vWF levels and stroke risk on genomic levels. Finally, (4) studies of patients with primary disorders of excess or deficiency of function in the vWF axis (e.g. thrombotic thrombocytopenic purpura and von Willebrand disease, respectively) which demonstrate the crucial role of vWF in atherothrombosis. Therapeutic inhibition of VWF by novel agents appears particularly promising for secondary prevention of stroke recurrence in specific sub-groups of patients such as those suffering from large artery atherosclerosis, as designated according to the TOAST classification.

## Dimensions of Stroke Burden

Stroke remains among the top three diseases causing death and disability worldwide. 1 In the United States, approximately 795,000 strokes occur each year, with an estimated incidence of 247/100,000 patient years. 2 While in China, incidence rates are highest, with up to 584 events per 100,000, 3 incidence rates in Europe are lower, with men at higher risk (141/100,000) than women (95/100,000). 4 As four out of five stroke events are of ischaemic origin, this entity constitutes the main driver for the high burden of stroke. 2

While mortality rates are decreasing in both high- and low-income countries, morbidity rates in stroke survivors are still increasing over time. 5 This may suggest that while current treatment options are able to prevent death from stroke, they have only a limited impact on the preservation and/or recovery of full brain function after an ischaemic event and hence there continues to be a high rate of disability. Furthermore, due to the aging of the general population, the individual's life-time risk to experience a stroke increases because age is still the main non-modifiable risk factor for cerebral ischaemia.

Besides enormous personal burden, costs in the United States for post-stroke care are estimated to be about $100 billion each year, including direct and indirect costs. 6 Within Europe, costs are estimated to be about $27 billion annually, accounting $18.5 billion for direct costs and $8.5 billion for indirect costs. 7

Many promising options for prevention and treatment of ischaemic stroke have been tested, including invasive endovascular techniques and conservative medical management strategies, but only a limited number of treatments have gained approval by the Food and Drug Administration and the European Medicines Agency for either stroke prevention and/or treatment within the last decade.

Development of pharmacotherapy for ischaemic stroke indications continues to lag behind than that for myocardial infarction. Especially in regard to anti-platelet therapy, many drugs with a favourable benefit:risk ratio in coronary disease have not proven beneficial in cerebrovascular disease, where the intrinsic susceptibility to treatment-induced bleeding is seemingly increased. These include: heparin, the GPIIb/IIIa inhibitors abciximab and tirofiban, 8 9 newer P2Y12-receptor blockers such as prasugrel 10 and the thrombin-receptor antagonist vorapaxar. 11 There is a particular unmet need in anti-thrombotic pharmacotherapy for secondary stroke prevention, which currently only includes aspirin or aspirin–dipyridamole and possibly in some circumstances clopidogrel.

Improved understanding of ischaemic stroke pathogenesis at the molecular level and the identification of high-risk sub-types of stroke should enable development of novel and specific treatment strategies. In recent years, von Willebrand factor (vWF) has been consistently implicated in the epidemiologic literature as a risk factor for ischaemic stroke. Its role in platelet thrombogenesis at sites of arterial vascular injury in the presence of high shear force, and functional ‘silence’ in other vascular beds in the absence of shear force, makes it a logical target for primary and secondary stroke prevention, and possibly also for stroke treatment.

This review summarizes available evidence for the rationale for vWF inhibition in ischaemic stroke in the light of disadvantages of currently used pharmacotherapy, suggests how these may be addressed by targeting the GPIb-vWF axis and discusses stroke populations that may benefit from vWF-targeted therapy. Animal studies that improved understanding of the impact of vWF on stroke have recently been thoroughly reviewed by Denorme and De Meyer 12 and thus will not be discussed in detail.

## Pathomechanism of von Willebrand Factor in Stroke

Ischaemic brain injury arises in conditions of insufficient blood supply. This results from thrombosis, emboli or systemic hypo-perfusion, which together account for approximately 80% of all strokes. Specifically, large artery atherosclerosis (LAA, per the Trial of ORG 10172 in Acute Stroke Treatment [TOAST] classification 13 ) accounts for 16% of all ischaemic strokes in the United States, 14 and for 25% in China. 3 Atherosclerotic lesions cause obstruction, which further promotes thrombus formation and embolization—resulting in reduced cerebral blood flow.

An important player in the propagation of thrombus formation and atherosclerosis is the blood glycoprotein vWF, which functions as a molecular bridge linking platelets and sub-endothelial collagen after vascular injury and furthermore serves as a chaperone for factor VIII (FVIII). VWF protects FVIII from proteolytic inactivation by activated protein C and S. 15 Therefore, low vWF levels are accompanied by low FVIII levels, which in combination exaggerate a bleeding tendency.

Endothelium-derived vWF is synthesized mainly within endothelial cells. After several post-translational modifications including cleavage of a propeptide, both mature vWF and the propeptide are stored in Weibel–Palade bodies. 16 In response to inflammation, 17 histamine, 18 thrombin, 17 19 20 fibrin or to exogenous desmopressin, 21 Weibel–Palade bodies fuse with the endothelial membrane and release ultra-large vWF multimers and its cleaved propeptide. While the latter is intra-cellularly required for multimerization and storage of vWF, its function in the bloodstream is not entirely clear. 22 Upon release, ultra-large vWF strands are cleaved by ADAMTS13 into smaller multimers, which circulate inactively in a coiled conformation in plasma. Exposure to sub-endothelial collagen after vascular damage uncoils and thereby activates vWF multimers, exposing their GP1b-binding sites and facilitating platelet adhesion. The higher the shear rate, the more successfully the unfolding process proceeds resulting in a size-dependent globule-stretch transition. 23 This underlines the role of vWF in atherothrombosis, where obstructions cause high blood velocity and shear.

Thrombus initiation by vWF–GPIb interactions is a crucial step, because it promotes platelet-rolling on the injured vessel wall and further engagement of platelet receptors and their substrates. One of these platelet receptors is GPIIbIIIa, which is not only capable of binding to fibrinogen, but also to vWF itself. However, while vWF–GPIb interactions are substantial for thrombus initiation, vWF-collagen and vWF-GPIIbIIIa mainly contribute to thrombus growth. 24

vWF has also been linked to numerous pathological processes including angiogenesis, cell proliferation and inflammation. Several mechanisms are involved in vWF-mediated inflammation, specifically platelet interactions and the GPIb–vWF axis. Direct interactions at low shear rates between vWF and leukocytes, as well as vWF-induced increased vascular permeability, diapedesis of leukocytes and accumulation of vWF in the extra-vascular space all promote inflammation. 25 Further potentiation of inflammation can result from low ADAMTS13 activity, as ultra-large vWF strings work in concert with P-selectin to promote platelet aggregation and platelet-induced leukocyte recruitment.

Inflammation plays a major role in atherothrombosis, highlighting its importance in ischaemic stroke. Arteriosclerosis is a localized inflammatory process in which increased amounts of ultra-large vWF strings are released from Weibel–Palade bodies under shear stress and attached to the inflamed endothelial surface. Cholesterol-induced atherosclerotic plaques were shown to contain high levels of Weibel–Palade bodies and vWF deposits. 26 27 Activated platelets trapped by endothelial vWF amplify the inflammatory process by recruiting leucocytes, macrophages and further platelets which in turn trigger the development of large vWF strings. 28 Progressing plaque formation increases shear stress, which again promotes further exocytosis of endothelial Weibel–Palade bodies. Due to reduced synthesis and increased thrombin cleavage, ADAMTS13 activity is impaired under inflammatory conditions facilitating the subsequent development of hyperactive vWF multimers. 29 In addition, vWF was shown to directly stimulate the proliferation of smooth muscle cells, a crucial component of arteriosclerotic lesions. 30 However, while vWF deficiency protects mice from atherosclerosis, 30 this protective effect has not been demonstrated in patients with von Willebrand disease. 31 Results from murine models must always be considered in the light that mice are not men. As wild-type mice are relatively resistant to atherosclerosis, ApoE knockout mice are most frequently used. With this genetic modification, vessel stenosis are more likely related to a process of angiogenesis and not to unstable atherosclerotic plaque formation, 32 which at least partly limits the translation of results from murine models into humans.

It is conceivable that pharmacological vWF inhibition may limit or even prevent development of atherothrombosis at vascular sites of high shear stress, but further studies are needed to conclusively define the potential of vWF as therapeutic target in the prevention or treatment of arteriosclerosis per se *.*

## The Role of von Willebrand Factor in Stroke—Insights from Epidemiological Studies

As vWF is critically involved in platelet aggregation and thrombus formation upon endothelial activation, it has been studied intensively as a potential risk factor for stroke and stroke-related outcome.

An association between vWF levels, the risk of first-ever ischaemic stroke, 33 34 stroke recurrence, 35 stroke severity, 36 37 post-stroke morbidity 38 39 and mortality 40 has been reported by several studies ( Table 1 ; Supplementary Table S1 and S2 , available in the online version). Many of them were observational and case–control studies, which investigated vWF levels by use of various assays at the time of the acute ischaemic event.

**Table 1:** Longitudinal studies on the association between vWF and ischaemic stroke

Reference	Sample size	Study name	Follow-up + new events	Association	Results	Adjusted OR/RR + covariates
**Longitudinal studies**						
Smith et al 55	1,592 subjects 55–74 y	Part of ‘Edinburgh Artery study’	5 y Total vascular events: 268 Coronary disease: 258 Stroke events: 45	vWF:Ag and risk of stroke by 1 SD increase	RR: 1.27 (CI, 0.95–1.72)	RR: 1.15 (0.85–1.57)
					Adjustments: age and sex	Adjustments: age, sex, systolic blood pressure, LDL cholesterol, smoking status and baseline disease
Folsom et al 34	14,713 subjects 45–64 y	Part of ‘Atherosclerosis Risk in Communities study’	6–9 y IS events: 191	vWF:Ag and risk of IS by 1 SD increase	RR: 1.36 (1.2–1.5)	RR: 1.26 (1.12–1.43) RR in highest vWF:Ag quartile: 1.71 (1.1–2.7)
						Adjustments: age, sex, community, race (except where stratified), systolic blood pressure and anti-hypertensive medication status (except where stratified), left ventricular hypertrophy, diabetes, HDL cholesterol, LDL cholesterol, waist-to-hip ratio, education and smoking status and amount
Smith et al 184	2,398 males 49–65 y	Part of ‘The Caerphilly study’	13 y Total vascular events: 486, Coronary disease: 353 IS events: 133	vWF:Ag and risk of IS in highest tertile	HR for IS: 0.98 (0.62–1.56)	HR: 0.97 (0.61–1.56)
					Adjustments: age	Adjustments: age, smoking status, diabetes, systolic blood pressure, total cholesterol, HDL cholesterol, total triglycerides, body mass index and family history of premature coronary heart disease
Ohira et al 88	14,448 subjects 45–64 y	Part of ‘Atherosclerosis Risk in Communities study’	13 y IS events: 531 (105 lacunar, 326 non-lacunar and 100 CE)	vWF:Ag and risk of IS sub-types by 1 SD increase	RR: - lacunar stroke: 1.21 (1.03–1.42) - non-lacunar stroke: 1.27 (1.16–1.39) - cardioembolic stroke: 1.58 (1.34–1.81)	RR: - lacunar stroke: 1.10 (0.93–1.29) - non-lacunar stroke: 1.20 (1.09–1.31) - cardioembolic stroke: 1.47 (1.28–1.70)
					Adjustments: age, sex and race	Adjustments: age, sex, race, waist-to-hip ratio, systolic blood pressure, smoking status, anti-hypertensive medication, diabetes mellitus, history of chronic heart disease, left ventricular hypertrophy, education level, HDL cholesterol, lipoprotein(a) and white blood count
Tzoulaki et al 56	1,592 subjects 55–74 y	Part of ‘Edinburgh Artery Study’	17 y Cardiovascular events: 416	vWF:Ag and CVD in highest tertile	HR: 1.42 (1.09–1.85)	HR: 1.33 (1.02–1.74)
					Adjustments: age and sex	Adjustments: age, sex, sub-clinical disease, pack-years smoking, diabetes, BMI, total/HDL cholesterol, physical activity and history of CVD
Wieberdink et al 185	6,250 subjects ≥55 y	Part of the ‘Rotterdam Study’	5 y IS events: 197	vWF:Ag levels and IS by 1 SD increase	HR: 1.13 (0.99–1.29)	HR: 1.12 (0.98–1.27)* HR: 1.10 (0.95–1.26)†
					Adjustments: age and sex	Adjustments: *adjusted for age, sex, systolic blood pressure, diabetes mellitus, total cholesterol, HDL cholesterol, lipid-lowering medication, smoking status, waist-to-hip ratio, atrial fibrillation, coronary heart disease, peripheral arterial disease and anti-thrombotic medication; †adjusted for all mentioned above and ABO blood group
Wannamethee et al 186	3,358 men 60–79 y	Part of ‘The British Regional Heart Study’	9 y IS events: 187	vWF:Ag levels and IS by 1 SD increase	HR: 1.24 (1.08–1.43)	HR: 1.25 (1.09–1.45)* HR: 1.18 (1.02–1.38)†
					Adjustments: Age	Adjustments: *age, smoking status, alcohol intake, body mass index, social class, physical activity, forced expiratory volume in 1 second, prevalent angina, diabetes, use of anti-hypertensive treatment and systolic blood pressure; †adjusted for all mentioned above and CRP
Sonneveld et al 54	5,941 subjects ≥55 y	Part of the ‘Rotterdam Study’	11 y IS events: 306	-Lowest versus highest quartile ADAMTS13	- HR: 1.61 (1.15–2.26)	- HR: 1.65 (1.16–2.32)
				-Per 1 SD decrease	- HR 1.18 (1.04–1.33)	- HR: 1.19 (1.05–1.34)
				-Lowest quartile ADAMTS13 activity + highest quartile VWF:Ag levels and IS	- HR: 1.72 (1.20–2.47)	- HR: 1.71 (1.19–2.45)
					Adjustments: age and sex	Adjustments: all: age, sex, anti-thrombotic medication, anti-hypertensive drugs, diabetes mellitus, lipid-reducing agents, BMI, smoking, total cholesterol, HDL cholesterol, systolic blood pressure and diastolic blood pressure

Abbreviations: Ag, antigen; BMI, body mass index; CaVD, cardiovascular disease; CE, cardioembolic; CI, confidence interval; CRP, C-reactive protein; CVD, cerebrovascular disease; HDL, high-density lipoprotein; HR, hazard ratio; IS, ischaemic stroke; LDL, low-density lipoprotein; OR, odds ratio; RR, relative risk; SD, standard deviation; TIA, transient ischaemic attack; vWF, von Willebrand factor.

Note: We searched Pubmed up to January 1, 2018 using the keywords ‘vWF’, ‘ischaemic stroke’, ‘risk’ and ‘outcome’ and reviewed previous studies investigating the association between vWF:Ag and pp levels as well as vWF activity measurements and the risk of and outcome in ischaemic stroke. We identified 8 longitudinal studies, which are summarized in this table.

Several laboratory tests are available for assessing vWF activity and vWF quantity ( Table 2 ). Measurements for vWF activity comprise the ristocetin cofactor assay (vWF:RCo) and collagen-binding assay (vWF:CB). The observation that ristocetin induces platelet aggregation led to the development of the first quantitative assay to determine vWF activity. Ristocetin alters vWF confirmation which provides the exposure of GPIb binding sites to facilitate platelet aggregation. Therefore, the vWF:RCo assay reflects the interaction between vWF and its cognate platelet receptor GPIb. Collagen-binding provides another essential part of platelet plug formation and therefore collagen-binding assays reflect vWF activity as well. vWF:CB may be useful as companion to vWF:RCo assays to reduce error rates due to test variability and sensitivity. 41 However, with respect to vWF in stroke, to date only vWF:RCo tests have been used to determine vWF activity.

**Table 2:** Laboratory methods to determine von Willebrand factor levels

	Measurement	Principle	Method	Advantage	Disadvantage
vWF antigen (vWF:Ag) a	Quantity	Antibody captures protein	ELISA, LIA or flow cytometry	High availability in laboratories	Different cut-off values for different assays Not sensitive for low levels No differential sensitivity to variable molecular weight forms of vWF
vWF propeptide (vWF:pp) a	Quantity	Antibody captures protein	ELISA	Sensitive parameter for vWF release	Different cut-off values for different assays Little commercial availability
vWF ristocetin co-factor (vWF:RCo) a	Activity	Ristocetin induces platelet aggregation through GPIb–vWF interaction	Aggregometry, standard coagulation instruments, LIA or flow cytometry	High availability in laboratories Availability at the bedside with timely results	High variation of coefficients Limited inter-laboratory reproducibility and standardization Poor sensitivity for low vWF levels
vWF collagen binding (vWF:CB)	Activity	Collagen induces vWF binding	ELISA or flow cytometry	Sensitivity to high molecular weight vWF Sensitivity to low vWF levels Little variability	Lack of comparative data
vWF activity	Activity	Recombinant gain of function GPIb binds to vWF	LIA or ELISA	Easy performance and automation High assay accuracy and precision Detection of vWF at low levels	Lack of comparative data

Abbreviations: ELISA, enzyme-linked immunosorbant assay; GPIb, glycoprotein Ib; LIA, latex-particle immunoassay; vWF, von Willebrand factor.

Note: Key points for different assessment of von Willebrand factor.

a Indicates tests used in stroke studies.

vWF can be measured quantitatively by either vWF:Ag or vWF:pp. As mature vWF and vWF propeptide differ in their kinetics, the assessment of both helps in determining vWF secretion, clearance and survival. The half-life of vWF propeptide is 2 to 3 hours and therefore shorter than the 8 to 10 hours half-life of mature vWF. 42 Furthermore, vWF propeptide is independent of blood type and ADAMTS13 activity, making it a more sensitive marker for vWF release. To date, only few studies investigated both vWF:Ag and vWF:pp levels, probably reflecting the wider availability of vWF:Ag assays. However, when interpreting results from stroke studies investigating vWF levels, disadvantages of the used methods need to be taken into account for the distinct method ( Table 2 ).

### Elevated vWF Levels in Stroke: Cause or Consequence?

Supportive evidence for a causative role of vWF in stroke comes from animal models, which have consistently shown that deficiency in vWF is protective against cerebral ischaemia. 43 44 Likewise, patients with von Willebrand disease are known to have a reduced risk of arterial thrombosis. 45 Conversely, observations in acquired thrombotic thrombocytopenic purpura patients underline the crucial role of vWF in stroke, as hereby the accumulation of ultra-large vWF multimers due to an autoantibody-induced decreased activity of ADAMTS13 leads to thrombotic complications such as stroke or myocardial infarction. 46

Numerous case–control studies that determined vWF levels at the time of ischaemic stroke showed a significant increase in vWF as compared with controls. 33 37 47 48 49 50 51 52 However, due to the nature of their study design, no assumptions on the cause–effect relationship can be made, i.e., whether elevated vWF levels reflect a risk of developing first-ever stroke or simply mirror inflammation and an activation of the haemostatic system and are therefore a consequence of acute ischaemia. For example, vWF levels correlate with C-reactive protein (CRP) levels on admission; 37 however, data are conflicting concerning persisting elevation of vWF in the late phase after stroke. 33 49 51 52

Markers closely associated with vWF, such as ADAMTS13 and FVIII, are also significantly different between stroke patients and controls; e.g., higher FVIII levels 47 and lower ADAMTS13 activity are found in stroke patients. 51 While there are no data on FVIII levels in the late phase after stroke, ADAMTS13 activity increases to normal levels. 51

Still, low ADAMTS13 activity might be an independent risk factor for stroke occurrence as shown by a large observational case–control study in young women with stroke in which the ADAMTS13 measurements were performed in the very late phase after stroke. The retrospective odds ratio (OR) for first-ever stroke comparing the lowest quartile to the highest quartile of convalescent ADAMTS13 activity was 3.1 (1.6–5.8), even after adjustment for several risk factors. 52 The combination of high vWF levels and low ADAMTS13 activity are the most crucial haemostatic factors for developing stroke. 53 54

Evidence for the causative role of vWF in stroke comes from large longitudinal studies that investigated stroke-naïve patients and analysed their stroke prevalence with respect to baseline vWF levels and ADMTS13 activity ( Table 1 ). Smith et al demonstrated a trend to an increased stroke-risk according to vWF levels in ‘the Edinburgh study’. They prospectively determined vWF levels in almost 1,600 stroke-naïve participants at baseline with a follow-up for cardiovascular events after 5 years. The age- and sex-adjusted relative risk was nominal 1.27 (confidence interval [CI], 0.95–1.72; for every unit increase in vWF-antigen level on a logarithmic scale), but this was not statistically significant. 55 In the same study cohort after a prolonged follow-up period of 17 years, the number of strokes in the population quadrupled and then the association of stroke risk and vWF antigen level became statistically significant, with the hazard ratio (HR) adjusted for age and sex of 1.42 (CI, 1.09–1.85) comparing top versus bottom tertile of vWF antigen. 56

In a much larger sample size, the ‘Atherosclerosis Risk in Communities study’ showed a positive association between baseline vWF levels and the risk of stroke occurrence. In this prospective study that followed more than 14,000 patients free of cardiovascular disease for up to 9 years, the risk of stroke in patients in the highest vWF quartile was 1.7 (CI, 1.1–2.7) times higher than in those in the lowest vWF quartile. Likewise, in an observational case–control study of patients with first time stroke, the risk of stroke increased by 80% with every quartile of vWF determined at the time of ischaemic event. 33 This finding might indicate that there could be a certain threshold level at which vWF becomes detrimental.

Further supporting data come from the ‘Rotterdam study’, another prospective population-based cohort study that followed more than 6,000 patients, who were free from stroke at baseline over a median follow-up time of more than 10 years (56,403 total person-years).

Increasing vWF levels were associated with a significant increase in the risk of stroke (HR per standard deviation increase: 1.12 [CI, 1.01–1.25]). In a subsequent analysis of the Rotterdam study population, Sonneveld et al were the first to perform a combined analysis for vWF-antigen and ADAMTS13 activity in a longitudinal study. Patients with high vWF Antigen levels (≥ 75th percentile) along with low ADAMTS13 activity (≤ 25th percentile) were at a significantly increased risk for ischaemic stroke with a HR of 1.71 (CI, 1.19–2.45; absolute risk, 9.1%). 54

Taken together, these data support the rationale that not only vWF levels, but also the vWF-modifying enzyme ADAMTS13 plays an important role in the development of first-ever stroke. Therefore, screening patients free of stroke at preventive check-ups could help to identify high-risk patients, stratifying them for risk of future stroke based on their vWF levels and/or ADAMTS13 activity.

### vWF Levels, Stroke and Genetics: All a Hereditary Risk?

Recent genome wide association studies identified several gene loci linked to sub-types of ischaemic stroke, 57 58 59 60 61 62 and genetic polymorphisms have gathered increased attention as tools for stratifying stroke risk. Genetics impact the risk of first-ever stroke, stroke recurrence and recanalization rates. 35 63 64

vWF plasma levels are strongly heritable 65 and determined by several genetic factors 66 67 68 69 70 including the ABO blood group, 71 72 73 74 75 76 77 vWF gene variants and single nucleotide polymorphisms. 76 78 79 80 81 The plasma concentration of vWF in individuals with blood group 0 is approximately 25% lower compared with those with types non-0. Therefore, the question arises whether the latter are at higher risk to develop ischaemic events. Data support this rationale in myocardial infarction, 82 but so far data from stroke patients did not confirm this hypothesis. Despite 25% lower vWF levels in type 0 individuals, the stroke risk was higher in patients in the highest vWF quartile with blood group 0 as compared with non-0. Yet, the mechanism behind this is unclear. 33

Genetic determinants and vWF levels were investigated with respect to overall stroke risk in the large prospective Rotterdam study. However, no genetic predictor of stroke risk was found while higher genetic scores correlated with vWF levels. 63 The authors therefore concluded that genetic variants may still determine cardiovascular and stroke risk in younger individuals, but that this trend was not demonstrated in the elderly Rotterdam study population. 83 However, variants of the ABO locus, which is an important determinant of vWF plasma levels, 35 63 71 72 were associated with large-vessel and cardioembolic but not small-vessel stroke. 84 Given the importance of thrombotic factors in the former two stroke sub-types, it seems rational that vWF and its genetics play an important role in these sub-types of stroke.

### Assessment of vWF to Predict Outcome and Stroke Recurrence

Data for the impact of vWF levels on stroke outcome and the risk for stroke recurrence came from observational and case–control studies. vWF levels were measured at the time of first ischaemic event and therefore no clear assumption can be made if vWF determines post-stroke morbidity and mortality as well as recurrence or only predicts outcome in stroke patients.

In this context, Carter et al found that 3-year mortality from stroke increases with vWF activity ranging from 16% in those with levels < 135% (normal = 100%) to 52% in patients with vWF levels > 243%. 40 Those who died within 30 days (∼5%) had total arterial circulation infarction more frequently as compared with patients dying after 30 days. Further data from the same study population showed that with every unit increase in vWF levels on a logarithmic scale, the risk of 6-month mortality increased by 73%. 47 Two recent smaller observational studies confirmed the association between high vWF levels and poor outcome. 36 38 Follow-up in these studies using disability scores was only until discharge and therefore increased vWF levels might reflect activation of inflammation and only mirror the extent of ischaemia.

With respect to stroke recurrence, few data are available. Williams et al showed that elevated vWF levels increase the risk for stroke recurrence in both unadjusted ( *p* = 0.0007; HR, 1.26) and fully adjusted ( *p* = 0.018; HR, 1.19) models. Tools to predict early stroke recurrence integrate only clinical and/or imaging information, but accuracy might improve taking laboratory parameters into account. 85 86 Therefore, vWF might serve as a potential candidate given its important pathophysiologic role as acute phase protein and in LAA, where stroke recurrence is deemed to be highest. We still lack confirming data, but given the fact that stroke recurrence is highest shortly after stroke, it would be desirable to gain new data on early stroke recurrence with respect to vWF levels.

### The Role of von Willebrand Factor according to Stroke Sub-Type

vWF levels are significantly higher in large- than in small-vessel disease. 47 49 High levels of vWF in LAA ( *defined above* ) sub-type of stroke compared with other sub-types have also been reported by Sonneveld et al in a large observational study. These authors additionally found a strong association between vessel calcification volume in the aortic arch and carotid arteries and plasma vWF levels at the time of ischaemic event in 925 patients. 87 Longitudinal assessments further confirmed these associations between vWF and the development of atherosclerotic-driven stroke, suggesting a causative role of vWF in specific stroke sub-types. 88

Therefore, these findings suggest that the LAA sub-type of stroke could be particularly sensitive to a vWF blocking strategy. This is plausible considering the key role of vWF in shear stress-dependent haemostasis at the site of vessel stenosis and plaques. 31 This may be especially relevant with respect to patients with carotid stenosis, who are known to have a high risk of stroke recurrence, as they have persistently elevated vWF levels after the first ischaemic event, 52 which may explain their high risk of stroke recurrence despite the use of conventional anti-platelet drugs. Of note, platelet function measured under high shear stress is minimally affected by conventional anti-platelet drugs including aspirin, 89 clopidogrel, 90 prasugrel 91 or tirofiban 92 in the presence of high vWF levels. Furthermore, it is unclear, whether vWF levels are altered by initiation of anti-platelet therapy with aspirin or clopidogrel per se. 93 94

## Shortcomings of Current Stroke Prevention and Treatment

### Limitations of Acute Ischaemic Stroke Treatment

To date, the only Food and Drug Administration-approved medication for acute ischaemic stroke is recombinant tissue plasminogen activator (rtPA), based on the results from the National Institute of Neurological Disorders and Stroke (NINDS) landmark study published in 1995. 95 Since then no single new medication has gained approval. Twelve trials have been published so far that compared the effects of thrombolytic agents and placebo of which only two showed positive outcomes: the NINDS part II study and the ECASS-III trial. 95 96 Still, in a meta-analysis of 12 randomized trials, treatment with rtPA significantly decreased mortality and disability, especially if administered within 3 hours after symptom onset. 97

More recently, mechanical endovascular interventions have been introduced into the standard of care for acute stroke management, and in 2015, the current guidelines for acute stroke treatment were updated and mechanical treatment with or without rtPA is recommended. Stent retrievers are recommended in addition to rtPA in internal carotid artery or middle cerebral artery-M1 occlusion. 98

#### The Issue of Time Delay

Intravenous thrombolysis is only administered to 5.2% 99 100 of all ischaemic stroke patients in the United States and 1.2% of Chinese patients. 101 The most critical criterion for exclusion is the time window of < 4.5 hours from stroke onset to treatment initiation. But still the majority of the patients who do arrive within the time window still do not receive rtPA for various reasons: because their stroke is not deemed severe enough, their symptoms improve rapidly or they have comorbidities such as hypertension that are believed to increase the risk of intra-cranial haemorrhage. 102 103 104 In developing countries, insufficient time to complete imaging studies and intra-hospital delay is another particularly critical issue. 101 The actual proportion of patients eligible for thrombolysis is very difficult to establish, as many co-factors need to be taken into account when interpreting thrombolysis rates. For example, thrombolysis rates differ between specialized centres that receive referral patients pre-determined to be eligible for thrombolytic treatment and smaller less specialized hospitals treating ‘all comers’. In several countries and federal states, thrombolysis proportions have reached 15 to 20% in specialized referral stroke treatment centres.

Although there is some evidence that mechanical thrombectomy is beneficial in a time window of up to 12 hours after symptom onset, i.e., when rtPA is not further indicated, definitive data are still lacking. However, a recent randomized controlled trial showed that thrombectomy was still beneficial if performed 6 to 24 hours after stroke onset in patients with a mismatch between infarct size and clinical disability. Thirty-three per cent more patients achieved functional independence by 90 days (modified Rankin Scale score 0–2) in the arm receiving thrombectomy plus standard medical care compared with those in the arm receiving standard medical care alone. As the sample size was rather small with 206 enrolled patients, larger trials confirming this benefit are still warranted. 105

#### Risks Attributed to Thrombolysis

In patients with myocardial infarction and pulmonary embolism who receive thrombolysis with rtPA, the risk for symptomatic intra-cranial bleeding is 1 to 1.5%. 106 However, in patients with stroke receiving rtPA the bleeding risk is 5 to 8% and increases up to 13% in those who re-canalize late. 106 The odds of intra-cranial haemorrhage double with co-morbidities such as atrial fibrillation, congestive heart failure and renal impairment, as well as in those patients taking anti-platelet medication. 107 Recombinant tPA interacts with several proteins and receptors, thereby impairing blood–brain barrier integrity and increasing the susceptibility of neurons to excitotoxicity by modulation of N-methyl-D-aspartate (NMDA) receptors, which emerging data suggest is a major cause of neuronal death after ischaemia. 108

#### Limited Recanalization Rates

However, in the limited number of patients who receive thrombolytics, early recanalization 2 hours after the start of rtPA treatment is achieved in approximately 50% of patients, 109 varying between occluded vessel sites but with the highest success rate in middle cerebral artery-M1 occlusion. 106 This may reflect the responsiveness of different thrombus sub-types to thrombolysis. Fibrin-rich clots mainly arise from cardiac embolism and are more sensitive to thrombolysis than are vWF-rich clots, which in general result from atherothrombosis and large vessel disease, not cardiac embolism. 110 About one-third of patients achieve late recanalization, which is accompanied by poor outcome caused by longer ischaemia. 109

#### Open Questions for Endovascular Treatment

Besides clarity concerning the beneficial time window for mechanical clot retrieval, definitive evidence concerning the best endovascular treatment option is still lacking. To date, mechanical stent-retrievers are the most promising devices. 111 This method combines the advances of prompt flow restoration with stenting and mechanical clot retrieval. While three earlier trials investigating endarterectomy devices showed no benefit, 112 five trials performed between 2010 and 2015 111 showed a decrease in disability as well as an increase in functional independence in patients treated with an intra-arterial mechanical thrombectomy approach 111 ; rates of intra-cranial bleeding were comparable between patients receiving the intervention and those receiving conventional therapy. Mortality rates in all five positive trials trended favourably but did not differ significantly between the intervention and the control group, 15.3 and 18.9%, respectively. 111 Rates of other intervention-specific complications, e.g., perforation of vessels, increase when the physician performing the treatment has limited experience in this area. This restricts the ability to offer mechanical treatment options to stroke centres with high patient volume, as these have the requisite resources and expertise. 113

Furthermore, there are only limited data for combined treatment with mechanical thrombectomy and concomitant anti-platelet agents. In a prospective study, administration of tirofiban in addition to endovascular thrombectomy was safe and associated with lower odds of death. 114 Given the crucial role of vWF under high shear rates, an anti-vWF approach in addition to mechanical clot retrieval potentially could be the optimal therapeutic strategy to prevent vessel re-occlusion and positively affect outcome after the procedure.

### Limitations of Secondary Stroke Prevention

Besides modification of traditional risk factors for stroke, such as hypertension, diabetes mellitus, smoking, dyslipidaemia and physical inactivity, 115 platelet inhibitors are the cornerstones of secondary non-cardioembolic ischaemic stroke prevention. Recent guidelines recommend anti-thrombotic mono-therapy with aspirin, or with aspirin and extended-release dipyridamole, or with the adenosine diphosphate-receptor blocker clopidogrel as equivalent alternatives. 116 Furthermore, cilostazol, a phosphodiesterase inhibitor, showed significant benefit in Asian patients for the prevention of stroke and other serious vascular events. However, to date cilostazol has not gained approval for secondary stroke prevention and has not yet been tested in a non-Asian population. 117 With regards to the individual choice of anti-platelet therapy, there is insufficient evidence to support the use of one agent over another.

Two previous trials, however, reported greater benefit for secondary stroke risk reduction with aspirin plus extended-release dipyridamole than with aspirin alone 118 ; and the CAPRIE trial, which compared clopidogrel to aspirin in 19,185 patients with symptomatic vascular disease, found clopidogrel to be of minor superiority (relative risk reduction of 8.7% in favour of clopidogrel; CI, 0.3–16.5, *p* = 0.043) over aspirin for the intention-to-treat population regarding the primary combined end point of ischaemic stroke, myocardial infarction or vascular death. 119 However, in the stroke sub-population the relative risk reduction of 7.3% (CI, –5.7 to 18.7) was not significant ( *p* = 0.26).

While data from animal studies showed a possible neuroprotective effect of aspirin with or without dipyridamole, 120 this has not been shown for clopidogrel. 121 Comparing aspirin-dipyridamole to clopidogrel in stroke patients, the net risk of recurrent stroke is similar but still 9% in each group of the ProFess trial, which prospectively followed more than 20,300 patients over 2.5 years. 122 The use of clopidogrel was even associated with an increased severity of recurrent stroke in a post hoc sub-group analysis of that trial. 123 124 This may reflect the lack of a neuroprotective effect of clopidogrel. 121

Short-term dual anti-platelet therapy with aspirin and clopidogrel for 90 days is indicated only in high-risk patients with symptomatic intra-cranial large artery disease, as these patients are known to have a high risk of early stroke recurrence after first-ever transient ischaemic attack. Aspirin and clopidogrel in combination, however, should not be used for long-term stroke prevention because of a lack of superiority compared with clopidogrel alone together with a substantially increased risk of major bleeding events. 125 126

The critical issue in secondary stroke prevention is finding the right balance between sufficient anti-platelet effects and a low bleeding risk. Recent data focusing on major bleeding events showed that the cumulative 3-year major bleeding risk for stroke patients on anti-platelet therapy was overall 23%. Risk for ischaemic events despite anti-platelet therapy was even above 60%. 127 This is an unacceptably high event rate and therefore carefully selected patients might benefit from an individualized, more effective anti-platelet therapy with an agent that is less likely to promote bleeding complications.

#### High On-Treatment Platelet Reactivity

One reason for the limited potency of aspirin and clopidogrel in secondary stroke prevention may be mediated by haemostatic mechanisms of platelet activation and thrombus formation that are independent from platelets' cyclooxygenase and adenosine diphosphate receptor pathways. Twenty years ago, it was already demonstrated that shear-induced platelet aggregation mediated by exogenous or platelet-derived vWF cannot be inhibited by aspirin in vitro. 128

In a recent longitudinal observational study investigating platelet function by means of multiple electrode aggregometry in 624 stroke patients on anti-platelet therapy, 11% of patients with a stroke while on aspirin had high on-treatment platelet reactivity, and treatment with clopidogrel was accompanied by even higher proportions of unresponsiveness (36%), independent of the etiologic sub-type of stroke. 129 Rates of responsiveness to clopidogrel were not associated with CYP2C19 polymorphisms. In this context, Kunicki et al investigated the effect of aspirin on platelet function in 463 patients with stroke, transient ischaemic attack or acute coronary syndrome. 130 Aspirin non-responsiveness measured by Platelet Function Analyzer-100 significantly increased with vWF levels ( *p* < 0.001), which likely contributes to the resistance to the inhibitory effects of aspirin under high shear rates. 128

Clopidogrel's efficacy in preventing stroke likely depends on CYP2C19 genetic variants, which determine conversion of the inactive pro-drug to its active metabolite. Clopidogrel in addition to aspirin reduced the risk of recurrent stroke only in patients who did not carry CYP2C19 loss-of-function alleles in a large trial involving almost 3,000 patients with minor ischaemic stroke or transient ischaemic attack. 131 The increased risk of stroke in carriers of this allele (risk ratio, 1.92, CI, 1.57–2.35; *p* < 0.001) was confirmed by a recent meta-analysis, which analysed 15 studies with 4,762 stroke or transient ischaemic attack patients treated with clopidogrel. 132 Thirteen out of these 15 studies included Asian, mostly Chinese, patients ( *n* = 4,009, 84%). Another very recent study investigated the impact of glycaemic control on clopidogrel responsiveness in Chinese patients. Data showed that treatment with clopidogrel was beneficial only in non-carriers of CYP2C19 loss-of-function alleles who maintained good glycaemic control. 133 Comparable data for non-Asian patients are scant and direct empirical evidence for anti-platelet non-responsiveness in stroke recurrence is generally scarce as the measurement of platelet reactivity in stroke patients is not commonly performed in clinical practice. 129

## How Targeting vWF could Overcome the Pitfalls of Current Stroke Prevention and Treatment

### von Willebrand Factor Targeting in Acute Stroke

Several novel pharmacological approaches have been tested to overcome the difficulties of current stroke therapy, but with ambiguous results. Desmoteplase and de-fibrogenating agents were drug candidates promising to overcome the issue of rtPA-resistant thrombi which showed encouraging results in terms of reperfusion rates and disability, but still failed due to an increase in intra-cerebral bleeding. 134 Therefore, a new therapeutic approach that restores cerebral blood flow without increasing bleeding complications is a desirable goal not yet attained, and vWF-targeting strategies might meet this need. Supporting evidence for the mechanistic rationale of therapeutic vWF inhibition in stroke comes from pre-clinical studies investigating the pathophysiologic role of GPIb-vWF axis in cerebral ischaemia. 12 The contribution of vWF to the architecture of arterial thrombi formed under shear stress and their resistance to therapeutic dissolution has recently been illustrated in animal models of acute ischaemic stroke. While thrombi were resistant to conventional anti-thrombotic agents and fibrinolysis because of ‘protective’ vWF-platelet aggregates forming their external layer, disruption of GPIb–vWF interactions and platelet cross-linking by vWF inhibition dissolved thrombi and restored cerebral blood flow. 135 136 137 Furthermore, treatment with recombinant ADAMTS13 reduced neurotoxicity induced by rtPA; in a mouse model, recombinant ADAMTS13 in combination with rtPA significantly reduced infarct volume compared with mice treated with rtPA only, reflecting a neuroprotective effect largely mediated by specific NMDA receptor blockade reducing excitotoxic cell death. 138

As the GPIb-vWF axis is haemostatically important, primarily under high shear conditions, physiological haemostasis under low or moderate shear conditions should theoretically not be impaired by vWF blockade. This is in agreement with available safety data on serious bleeding events from pre-clinical and clinical studies investigating vWF inhibitors in healthy volunteers 139 and in patients with thrombotic thrombocytopenic purpura 140 141 142 and von Willebrand disease. 143 Stenotic cerebrovascular arteries leading to increased blood velocity and shear stress are pre-dilected sites for thrombus embolization and total occlusion with resulting cerebral ischaemia. While rtPA promotes thrombolysis within all blood vessels, vWF-targeting strategies should act selectively at stenotic, vWF-rich sites. Direct vWF-blocking agents showed promising results in pre-clinical studies. The nano-body, caplacizumab (ALX-0081), reduced brain infarct size in guinea pigs by GPIb-vWF blockade with no bleeding complications. 136 Furthermore, ADAMTS13-mediated cleavage of ultra-large vWF, which reduces adhesion of platelets and clot formation, has been assessed. Recombinant ADAMTS13 successfully restored cerebral blood flow and reduced cerebral infarct size up to 1 hour after occlusion with no increase in cerebral haemorrhage in a mouse model with rtPA-resistant, vWF-rich middle cerebral artery thrombi. 137 However, both studies only investigated drug administration up to 1 hour after occlusion—a time window that does not reflect a clinical setting and therefore the critical point of the narrow time window in stroke treatment has not been assessed by these studies.

### Sample Size Considerations in Targeting von Willebrand Factor in Primary Stroke Prevention

Recently, the large COMPASS trial, including more than 27,000 patients, showed a risk reduction for stroke when rivaroxaban was combined with aspirin versus aspirin alone, while rivaroxaban alone was not significantly better. 144 The low number of stroke events in this patient population despite pre-existent stable cardiovascular disease requiring high patient numbers to demonstrate benefit is not ideal for a first development program, as a sample size of more than 2 × 4,000 patients would be required to test the efficacy a new treatment such as an vWF inhibitor.

### Targeting von Willebrand Factor in Secondary Stroke Prevention

vWF might not only be an attractive target in acute stroke treatment but also in secondary stroke prevention, particularly if aimed at a sub-population at high risk of stroke recurrence that could especially benefit from a more potent anti-platelet therapy with a better risk profile.

While multiple agents representing several drug classes have proven benefit in coronary artery disease, including heparin, GPIIb/IIIa inhibitors, P2Y12-receptor antagonists, such as ticagrelor and prasugrel, as well as the thrombin-receptor antagonist vorapaxar, none of these drugs have improved a benefit:risk ratio for stroke prevention superior to aspirin.

#### Ticagrelor, Prasugrel and Vorapaxar Did Not Improve Outcome in Stroke Prevention

The use of ticagrelor, a more potent third-generation adenosine diphosphate-receptor blocker, was not superior to aspirin in reducing the rate of stroke recurrence, myocardial infarction or death within 90 days in a large prospective trial involving 13,199 patients with a non-severe ischaemic stroke or high-risk transient ischaemic attack, and thus is currently not recommended. 145 Nevertheless, sub-group analysis ( *n* = 3,906) suggest that Asian patients or those suffering from LAA could potentially benefit from the more potent platelet inhibition by ticagrelor. 146

Prasugrel is contraindicated in patients with a history of cerebrovascular events based on results from the TRITON-TIMI trial. This study compared prasugrel to clopidogrel in more than 13,600 patients with acute coronary syndrome and found a significant harm from prasugrel among those with a history of stroke or transient ischaemic attack. 10 Recent data from a Japanese study confirmed the lack of benefit of prasugrel in comparison to clopidogrel in 3,747 non-cardioembolic stroke patients. 147

Likewise, the thrombin-receptor antagonist vorapaxar is contraindicated in patients with prior stroke, because it increases the risk of intra-cranial haemorrhage in this population (HR, 2.52; CI, 1.46–4.36) 11 without improving the rate of ischaemic vascular events. However, more recent data suggest that vorapaxar may reduce the risk of primary ischaemic stroke in patients with atherosclerosis but without clinically evident cerebrovascular disease when used as mono-therapy rather than in addition to standard anti-platelet therapy aspirin or clopidogrel. 148

#### Dual Anti-Platelet Therapy

The use of dual anti-platelet therapy with clopidogrel and aspirin in secondary stroke prevention is considered beneficial only in the first 90 days because it was shown to cause harm in the form of major bleeding thereafter. 149 150

These data highlight the need for novel anti-thrombotic strategies that are safer and more effective than available anti-platelet agents, especially regarding *long-term* prevention of stroke recurrence. Aspirin reduced the risk of early recurrent stroke (i.e. in the first 6 weeks from the index event) in a recent longitudinal study investigating individual patient data from all available controlled trials on use of aspirin versus control in secondary prevention after transient ischaemic attack or ischaemic stroke, in contrast, its use had no effect on the 12-week risk of stroke recurrence. 123 Taken together, vWF-targeting agents could possibly overcome the pitfalls of current stroke therapy and prevention, but to date such agents have only been tested in animal models of stroke.

## von Willebrand Factor Inhibitors in Ischaemic Stroke and Target Populations

**Fig. 1:**
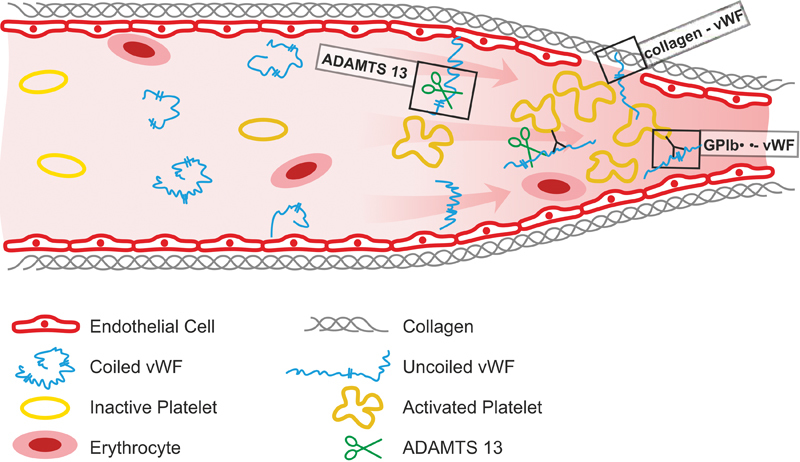
Scheme of von Willebrand factor (vWF) under high shear rates. Legend: Different mechanisms of actions of investigational von Willebrand factor inhibitors are described, i.e., GPIbα-vWF, ultra-large vWF multimers (recombinant ADAMTS13) and collagen-vWF. GP, glycoprotein.

There are three potential targets that mediate vWF-dependent thrombus formation. These are vWF-GPIb, vWF-collagen and vWF-GPIIbIIIa. However, animal models showed, that while collagen- and GPIb-binding are essential in stroke development, GPIIbIIIa interactions are without any relevance. 151 The insignificant effect of vWF-GPIIbIIIa binding in stroke is therefore compatible with the negative clinical trials of the GPIIbIIIa receptor inhibitor Abciximab. 8 Thus, only GPIb-vWF and collagen-vWF blockers are in the focus of drug development for stroke. In previous years, vWF inhibitors have been investigated in various pre-clinical and clinical studies ( Table 3 and Supplementary Table S3 , available in the online version). Several substances have been evaluated in animal models, of which five have been tested in humans. Molecules suitable for targeting vWF include antibodies, nano-bodies, aptamers and a vWF antagonist derived from snake venom. Mechanisms of action are described in Fig. 1 .

**Table 3:** Human studies of investigational von Willebrand inhibitors

Human studies					
Name	Type	Target	Underlying disease	Outcome	Reference
ARC1779	DNA/RNA aptamer	GPIbα-vWF	vWD type II	Desmopressin-induced thrombocytopenia	Jilma et al 143 Jilma-Stohlawetz et al 187
			Acquired and congenital TTP	Pharmacokinetics, pharmacodynamics, safety	Jilma-Stohlawetz et al 188 Cataland et al 142 Jilma-Stohlawetz et al 189
			Carotid stenosis	Inhibition of micro-embolic signals up to 3 h after carotid endarterectomy	Markus et al 155
AJW200	Monoclonal antibody	GPIbα-vWF	Healthy volunteers	Safety	Machin et al 190
rADAMTS13	Recombinant ADAMTS13	Ultra-large vWF multimers	Congenital TTP	Safety	Scully et al 191
Anfibatide	Snake venom derived	GPIbα-vWF	Healthy volunteers	Safety	Hou et al 192
ALX-0081 (caplacizumab)	Nano-body	GPIbα-vWF	Acquired TTP	Time to normalization of platelet count; number of exacerbations and relapses	Peyvandi et al 46

Abbreviations: GP, glycoprotein; TTP, thrombotic thrombocytopenic purpura; vWD, von Willebrand disease; vWF, von Willebrand factor.

Nano-bodies are fragments of antibodies with lower molecular mass and size, which enable better permeability and quicker elimination. Furthermore, nano-bodies are less likely to cause cytotoxicity triggered by complement activation, as they lack the Fc-region. 152 The first anti-vWF-nano-body will likely be approved for the treatment of TTP, due to positive results of recent Phase II/III trials. 46 153

Another approach to target specific molecules is facilitated by aptamer technology. Aptamers are molecules composed of single stranded nucleic acids folded into a specific tertiary structure, that specifically bind to their target, e.g., proteins, enzymes, viruses or receptors. 154 Methods used to produce aptamers are based on the principle of repetitive amplification and selection processes in vitro, producing molecules with high affinity. 154 In contrast to antibody production, chemical synthesis of aptamers does not require a biological system (e.g. Chinese hamster ovary cells) for production. Various in vitro studies have proven the capacity of anti-vWF aptamers in inhibiting vascular platelet adhesion only under high shear and one clinical trial has shown that an anti-vWF aptamer is capable of significantly reducing micro-embolic signals in the cerebral circulation immediately following carotid endarterectomy. 155

### Target Populations That May Benefit from von Willebrand Factor Inhibition

As vWF–platelet interactions are promoted by arterial shear stress, it is conceivable that patients with conditions, in which vWF likely plays a key role in thrombus formation, would in particular stand to gain benefit from therapeutic vWF blockade. This may include patients with stenotic LAA, those who have undergone carotid stenting, patients with persistently elevated vWF levels after an ischaemic event and those unresponsive to fibrinolytic or anti-platelet therapy.

Whether vWF is independently associated with non-responsiveness to conventional anti-platelet drugs across the heterogeneous population of ischaemic stroke patients or is rather a major contributing factor in specific stroke sub-types or in the presence of distinct co-morbidities is as yet unclear and needs further investigation.

The proportion of stroke patients unresponsive to aspirin and clopidogrel measured by whole blood aggregometry, however, is substantial. 129 Interference with platelet-activating pathways independent of cyclooxygenase and adenosine diphosphate receptors certainly constitutes one promising therapeutic target. In this context, the association between high vWF levels and aspirin unresponsiveness is interesting. 130 The question whether and to what extent vWF inhibition may overcome high on-treatment platelet reactivity in ischaemic stroke, however, has not yet been clearly answered.

Future studies need to define populations that are prone to developing vWF-mediated stroke and identify stroke sub-types that are sensitive to vWF inhibition to further refine the strategy for the clinical development of such agents.

#### Patients with Stroke Caused by von Willebrand Factor-Rich Thrombi

Early re-canalization and vessel re-opening is vital to achieving good outcome after ischaemic stroke. Attempts at vessel re-canalization by thrombolytics, however, fail in the large majority of patients. 106 Response to lysis and rates of early re-canalization by rtPA are particularly low in large vessel disease. One reason for this might be functional resistance of clots to therapeutic fibrinolysis. 110

Lytic susceptibility depends on the specific structural aspects of the clot. vWF-rich thrombi formed under shear stress were shown to be resistant to conventional recombinant tissue-type plasminogen activator in both stroke and myocardial infarction. 156 157 A substantial fraction of thrombi retrieved from stroke patients contain high levels of vWF. vWF-rich thrombi are resistant to conventional rtPA, but are however sensitive to vWF cleavage by early infusion of ADAMTS13. Higher baseline ADAMTS13 levels and activity > 75% in patients with acute stroke predict re-canalization success at 2 hours. 158 Blocking the GPIb-vWF axis on top of therapeutic plasminogen activation could be of therapeutic benefit because it may overcome lysis resistance in selected patients. In this context, the identification of vWF-rich thrombi by computed tomography or magnetic resonance imaging to guide treatment could become an interesting future approach for more individualized stroke therapy. 159

#### Patients with Large Artery Atherosclerosis

In patients with 50 to 99% stenosis of a large intra-cranial artery, the annual rate of ischaemic stroke ranges between 6 and 15% despite standard medical treatment with current anti-platelet agents. 160 161 Particularly, patients with severe (≥ 70%) or haemodynamically significant stenosis, those with ischaemic symptoms, and women with symptomatic intra-cranial arterial stenosis are at high risk of stroke. 160 162 Intra-cranial stenting is not beneficial in these patients according to the disappointing results from two large randomized trials. 161 163 Aspirin is usually used for long-term stroke prevention. In an ex vivo study on human atherosclerotic plaques, however, acetylsalicylic acid in contrast to a GPIb inhibitor failed to inhibit plaque-induced platelet thrombus formation under arterial flow conditions. 89 This may at least partly explain the continuing high rates of ischaemic stroke despite treatment with aspirin in patients with atherosclerotic stenosis of a large intra-cranial artery. 89 Given the high rates of ischaemic events in those patients, however, novel therapeutic strategies would be desirable.

Following results from a previous meta-analysis comparing incidence and sub-types of stroke between Chinese and European patients, the prevalence of LAA is significantly higher in Chinese patients. 3 Similarly, LAA sub-type of stroke was the most common stroke aetiology found in the Korean stroke registry. 164

Increasing insights into vWF-mediated platelet–endothelium interactions at the site of arteriosclerotic lesions suggest that patients with intra-cranial major artery arteriosclerosis could possibly benefit from (add-on) treatment with a vWF-blocking agent. Plaque-associated thrombus formation is critically dependent on blood-borne vWF, offering the opportunity for a vWF-targeting approach.

The significance of vWF in human atherogenesis, however, remains under investigation. High levels of vWF in LAA patients, 87 the strong association of vWF with the extent of atherosclerotic plaques and the inverse relation between plasma ADAMTS13 activity and the risk of both cerebro- and cardiovascular disease 54 165 reported by clinical studies yet suggest that vWF may serve a promising therapeutic target in large vessel disease.

In animal models of atherosclerosis, both the inhibition and deletion of vWF successfully reduced inflammation, plaque size and platelet adhesion, 166 167 168 while the deletion of ADAMTS13 had opposite effects. 169 170 Human clinical data on the impact of vWF inhibition on atherosclerosis are not available.

#### Secondary Prevention after Endarterectomy, Angioplasty and Stenting

Lifelong administration of aspirin is recommended after carotid endarterectomy. To date, endarterectomy with stenting is recommended only in patients with symptomatic extra-cranial internal carotid artery atherosclerotic disease without total occlusion. Restenosis, however, may occur in up to 12% of patients within the first 24 months. 171 Data from a 2003 Cochrane review furthermore suggest that the use of anti-platelet agents after carotid endarterectomy has no effects on all-cause death but may increase the risk of bleeding. 172

In patients who undergo carotid artery stenting, dual anti-platelet treatment is commonly administered for 6 weeks to limit the risk for thromboembolic complications and restenosis. 173 Evidence for this therapeutic approach, however, is yet limited and restenosis rates range between 0.5 and 5% for early and up to 7% at 10 years for late events. Early restenosis results from procedure-related vascular injury with subsequent intimal hyperplasia and occurs more frequently in women, diabetics and patients with neck irradiation. 174 175 176

Endovascular procedures induce damage and activation of the endothelial layer with subsequent release of inflammatory markers including vWF release, 177 178 which may contribute to the risk of early and late thromboembolic complications. Platelet activation during carotid endarterectomy even occurs despite the use of anti-platelet treatment. 179

The occurrence of stent restenosis was significantly associated with persistently elevated vWF levels in a prospective study investigating the relation between vWF plasma levels and restenosis after carotid artery stenting within the first 6 months. 180 It is conceivable, that additional vWF blockade on top of conventional anti-platelet therapy may provide superior anti-thrombotic effects at the site of endothelial damage after endarterectomy or stent placement by impairing localized vWF-driven platelet aggregation and subsequent thrombus formation. In this context, vWF inhibition by the aptamer ARC 1779 significantly reduced sonographically detectable micro-embolic signals within 3 hours after carotid endarterectomy in a clinical trial on 36 patients with carotid stenosis. 155 Likewise, in rat models, vWF inhibition significantly decreased platelet adhesion, intimal hyperplasia, stenosis and thrombosis after carotid endarterectomy. 181 Together, these data support a rationale of vWF blockade after endovascular procedures.

#### Asian Population

The rationale for why Asian individuals in particular might benefit from an anti-vWF approach is based upon several pieces of evidence. In China, approximately 2.4 million people suffer from stroke each year, 182 which constitutes the highest rate worldwide. Chinese patients have lower mean age at stroke onset 3 and it is especially noteworthy that intra-cranial atherosclerosis, a relatively rare form of cerebrovascular disease in Caucasians, is highly prevalent in young Chinese stroke patients. 183

This disproportionally high rate of intra-cranial atherosclerosis, the high prevalence of ischaemic stroke overall and the large percentage of LAA stroke sub-types 3 in Asian countries, makes Asian patients probably suitable for a more potent anti-platelet strategy. Furthermore, a sub-group analysis of outcome data in the SOCRATES study 146 suggested that platelet inhibition may translate into a more pronounced clinical benefit in Asians. Hence, Asians may represent a particularly suitable population to test the benefits of platelet inhibition by targeting vWF-mediated platelet adhesion and aggregation. In addition, the high number of CYP2C19 alleles carriers in the Chinese population 131 and the associated poor metabolic activation of and hence poor responsiveness to clopidogrel especially in patients with poor glycaemic control, support the rationale for a vWF-targeting approach.

## Conclusion

The rationale for vWF as a potential target in stroke comes from four bodies of evidence: animal models, that help us to understand the pathogenesis of stroke and give rise to the concept of vWF-targeting strategies; epidemiologic data demonstrating that plasma levels of vWF predict the occurrence and recurrence of stroke; genetic studies that establish an association between vWF levels and stroke risk on genomic levels; and lessons learned from patients with vWF disorders that showed the crucial role of vWF in atherothrombosis and stroke. However, randomized clinical trials with vWF inhibitors are needed to ultimately demonstrate the relevance of blocking vWF for the prevention or treatment of ischaemic stroke in this patient population.

Conceivably, identification of vWF-rich thrombi with high-resolution imaging techniques could help to stratify patients susceptible for an anti-vWF approach in the acute setting. Individualized acute stroke treatment could improve re-canalization rates with the maintenance of acceptable bleeding rates.

Also, for primary and secondary stroke prevention, targeting high-risk populations will likely improve the benefit:risk ratio. Therefore, sub-populations of patients need yet to be defined that might best respond to vWF inhibition. The contemporary understanding of stroke as a clinically and biologically diverse entity led to the consideration of a more individualized approach to pharmacotherapy and endovascular interventions for care of patients with stroke, based on risk stratification and careful differentiation between patient sub-groups. However, patients' risk stratification should probably not only be based on pathophysiologic mechanism but also on the genetic determinants and biomarkers that reflect increased risk. Specifically, patients with a high degree of LAA and CYP2C19 loss of function alleles carriers might be ideal candidates for a vWF targeting approach.
