# Supplementary material for: Targeting von Willebrand Factor in Ischaemic Stroke: Focus on Clinical Evidence
Source: Thromb Haemost. 2018 May 30;118(6):959–78. doi: 10.1055/s-0038-1648251 (PMC6193403; doi:10.1055/s-0038-1648251)
Supplement: Supplementary file 1 — Supplementary Material [file 10-1055-s-0038-1648251-s170843.pdf]

Supplementary Table S1 Studies on the association between vWF and ischaemic stroke

Reference (including PMID number)	Sample size	Population	Measurements + endpoint	Follow-up	Results
**Case control studies**					
Catto et al,<br>Thromb Haemost, 1997<br>9241740	208 patients<br>184 controls	Patients with stroke and healthy controls	vWF:Ag levels and FVIII:C initially and after 3 mo	6 mo	- vWF antigen and FVIII:C significantly higher in cases than in controls<br>- Initial vWF and FVIII:C levels significantly higher in subjects with LVD compared with the SVD group<br>- OR 1.73 (1.12, 2.66)* for death by increase of 1 U/mL in vWF levels
			Mortality<br>Association between vWF/FVIII and stroke subtype		Adjustments: *stroke sub-type
Qizilbash et al,<br>Neurology, 1997<br>9409345	95 patients<br>236 controls	Patients with TIA or minor IS and age- and sex-matched controls	vWF:Ag levels at admission	None	- vWF antigen significantly higher in cases than in controls<br>- OR for ischaemic stroke in highest quartile of vWF: 2.36*
			Association between vWF:Ag and ischaemic events		Adjustments: *age and sex
Bongers et al,<br>Stroke, 2006<br>16990571	124 patients<br>125 controls	First-ever IS patients and age- and sex-matched controls	vWF:Ag, vWF:RCo, ADAMTS13 and FVIII 7–14 d after stroke + vWF:Ag and vWF:RCo in 64 patients 3 mo after stroke	None	- vWF-Ag, vWF:RCo and FVIII:C initially significantly higher in cases<br>- OR in highest quartile of vWF:Ag: 3.2 (1.4–7.5)<br>- Other measurements not significant<br>- No difference in vWF:Ag and vWF:RCo 3 mo after stroke between cases and controls
			vWF:Ag levels, vWF:RCo activity, FVIII:C and ADAMTS13 activity and stroke risk		
Carter et al,<br>Stroke, 2007<br>17446429	545 patients<br>330 controls	IS patients and age-matched controls	Association between vWF:Ag, FVIII:C and death >30 d after events	7 y	- HR for highest quartile FVIII for death 2.06 (1.40–3.02)<br>- HR from univariate analysis for highest quartile vWF:Ag and post-stroke mortality: 3.92 (2.46–6.23)<br>- HR from multivariate analysis* for highest quartile vWF:Ag and post-stroke mortality: 2.15 (1.18–3.91)
					*Cox regression analysis including age, stroke sub-type, previous stroke/TIA and AF

(*Continued*)

Supplementary Table S1 (*Continued*)

Reference (including PMID number)	Sample size	Population	Measurements + endpoint	Follow-up	Results
Hanson et al,<br>J Thromb Haemost, 2011<br>21054779	600 patients<br>600 controls	Data from 'SAHLSIS';<br>First-ever or recurrent IS patients and age, gender and geographic area-matched controls	Association between vWF:Ag levels and stroke sub-type at admission and after 3 mo	3 mo	- vWF:Ag significantly higher in cases (both initially and after 3 mo) than controls<br>- vWF:Ag significantly higher in acute phase than after 3 mo in cases<br>- Significantly higher vWF:Ag levels in LVD and CE sub-type than SVD in acute phase; Significantly higher vWF:Ag in CE sub-type than SVD after 3 mo<br>- Unadjusted OR for acute phase vWF:Ag and overall IS: 2.00 (1.74–2.29)<br>- Adjusted OR for vWF:Ag and overall stroke: 2.01 (1.73–2.34)*; 1.73 (1.40–2.14)†
					Adjustments: *Smoking, diabetes, hypertension, and hyperlipidaemia for overall IS, as well as age, sex and geographic area for sub-types; †All as mentioned above and hsCRP and fibrinogen
van Schie et al,<br>J Thromb Haemost, 2010<br>20345720	171 patients<br>107 controls	'The ATTAC study', patients with first IS or TIA and controls, age 18–55 y	vWFpp and VWF:Ag levels at 3 mo	None	- vWF:Ag significantly higher in cases than in controls<br>- vWF:pp not significantly different<br>- RR for IS in highest VWF:pp and vWF:Ag quartile: 1.7 (95% CI, 1.1–2.8)* and 1.9 (95% CI, 1.1–3.1)*
					Adjustments: *Age, sex, smoking, hypertension, diabetes mellitus, hyperlipidaemia
van Schie et al,<br>J Thromb Haemost, 2010<br>20345720	101 patients<br>103 controls	'The COCOS study', patients with first IS or TIA patients and controls, age 18–75 y	vWFpp and vWF:Ag levels 7–14 d after the event	None	- vWF:Ag significantly higher in cases than in controls<br>- vWF:pp not significantly different<br>- RR for IS in highest vWF:pp and vWF:Ag quartile: 1.9 (1.0–3.6)* and 1.9 (1.0–3.3)*
					Adjustments: *Age, sex, smoking, hypertension, diabetes mellitus, hyperlipidaemia
Andersson et al,<br>Blood, 2012<br>22110247	175 patients<br>638 controls	'Ratio study', Frequency-matched case–control study of women and age, area of residence and year of event-matched female controls	Association between vWF:Ag and ADAMTS13 levels and risk of IS in young women; measurements median 95 mo after event	None	- OR for highest vWF:Ag quartile for IS: 6.7 (3.2–13.8)*<br>- OR for lowest quartile ADAMTS13 activity for IS: 3.1 (1.6–5.8)* for IS<br>- OR for combined lowest 10% ADAMTS13 activity and 10% highest vWF:Ag levels: 5.8 (1.7–20.2)*

Supplementary Table S1 (*Continued*)

Reference (including PMID number)	Sample size	Population	Measurements + endpoint	Follow-up	Results
					*Adjustments: Age, year of event/index year, area of residence, hypercholesterolemia, hyper-tension, diabetes mellitus and smoking
Kraft et al, PLOS One, 2014 24937073	233 patients 104 controls	Patients with TIA or IS and healthy controls	- Predictors of vWF:Ag levels at admission<br>- Association between VWF levels and key demographic and clinical characteristics<br>- Difference in vWF levels between acute and chronic CVD	None	- vWF:Ag levels significantly higher in cases than controls<br>- Independent predictors of vWF:Ag levels in multivariate analysis: NIHSS > 15 points, intake of platelet inhibitors before blood withdrawal and CRP at admission<br>- Significant association between CRP, leukocyte sub-sets, age, sex and stroke severity and vWF: Ag levels
McCabe et al, J Neurol Sci., 2015 25498844	53 patients 22 controls	Patients with TIA or IS on aspirin in the early phase ($\leq$ 4 wk) and in the late phase ($\geq$ 3 mo) and controls	vWF:Ag levels and ADAMTS13 activity in acute phase (< 4 wk) and $\geq$ 3 mo after the event	3 mo	-vWF:Ag significantly higher in acute phase and after 3 mo in cases than controls<br>- ADAMTS13 activity significantly lower in acute phase but not after 3 mo in cases than in controls
Tobin et al, J Neurol Sci., 2017 28320178	164 patients 27 controls	Patients $\leq$4 wk of TIA or IS and age- and sex-matched controls	VWF:Ag and VWF:pp levels at baseline ($\leq$4 wk), 14 and 90 d later	3 mo	-Significantly higher vWF:Ag and vWF:pp levels at all time points in cases than in controls<br>- Highest vWF:Ag and vWF:pp levels in LAA stroke compared with other stroke sub-types and significantly higher levels at all time points compared with controls

Abbreviations: AF, atrial fibrillation; CVD, cerebrovascular disease; FVIII:C, factor VIII concentration; HR, hazard ratio; hsCRP, high sensitive C-reactive protein; IS, ischaemic stroke; LAA, large artery atherosclerosis; LVD, large vessel disease; NIHSS, National Institute of Health Stroke Scale; OR, odds ratio; RR, relative risk; SVD, small vessel disease; TIA, transient ischaemic attack; vWF, von Willebrand factor; vWF:Ag, antigen; vWF:pp, propeptide; vWF:RCo, ristocetin co-activity.

Note: We searched Pubmed up to January 1, 2018, using the keywords 'vWF', 'ischaemic stroke', 'risk' and 'outcome' and reviewed previous studies investigating the association between vWF:Ag and pp levels as well as vWF activity measurements and the risk of and outcome in ischaemic stroke. We identified 11 case–control studies, which are summarized in this table.

Supplementary Table S2 Studies on the association between vWF and ischaemic stroke

Reference	Sample size	Key points	vWF-related endpoint	Follow-up	Results
**Observational studies**					
Sonneveld et al, Atherosclerosis, 2013 24075746	925 patients	Patients with TIA or IS	- Association between atherosclerosis and vWF:Ag levels<br>-Association between vWF levels and outcome	None	- Positive association between vWF:Ag levels and calcification volume<br>- Significantly higher vWF:Ag levels in LAA compared with other sub-types<br>- OR* for high vWF levels (> 1.43 IU/mL) compared with low for poor outcome: 1.45
					Adjustments: *Age, sex, blood group, cardiovascular risk factors, aortic arch and carotid calcification volume
Samai et al, Stroke, 2014 25028444	148 patients	Patients with IS from a prospective stroke registry	-Association between FVIII:C and vWF Activity and outcome/ adverse events at discharge	Until discharge	-OR* for combined elevated vWF activity (> 200%) and FVIII:C (> 200%) for poor functional outcome: 2.87 (1.17–7.06)<br>-OR† for combined elevated vWF activity (> 200%) and FVIII:C (> 200%) for:<br>Inpatient complications: 8.6 (1.57–46.85)<br>Neuroworsening: 3.21 (1.18–8.72)<br>Recurrent thrombotic events: 4.21 (1.57–11.28)
					Adjustments: *Age, NIHSS and glucose; †additional adjustment for tissue-type plasminogen activator
Menih et al, Wien Klin Wochenschr, 2017 28409234	108 patients	Patients with IS	Association between vWF Activity and stroke severity, clinical outcome and sub-type	None	- Multivariate analysis: vWF activity as predictor for NIHSS > 8 on admission<br>- Multivariate analysis: vWF activity as predictor for poor outcome at discharge (MRS ≥3)<br>- No difference of vWF activity between stroke sub-types
Williams et al, Stroke, 2017 28495826	2,100 patients	'The VISP trial', patients with IS	- Association of vWF activity and risk of stroke recurrence	2 y	- HR* for 1 SD increase in vWF for stroke recurrence: 1.19 ($p = 0.018$)<br>- HR for 1 SD increase in vWF for all-cause mortality: 1.21 ($p = 0.0122$)
					Adjustments: *Intervention group (high- or low-dose B vitamins), age, race, sex, smoking, body mass index, diabetes mellitus status, hypertension and low-density lipoproteins
Tobin et al, J Neurol., 2014 24781842	91 patients	Patients with TIA or IS	The impact of commencing or changing anti-platelet therapy on VWF:Ag and VWF:pp levels measured within 4 wk then 14 d (and > 90 d after changing anti-platelet therapy	3 mo	- No changes in VWF:Ag after commencing aspirin and after changing from aspirin to clopidogrel<br>- Significant reduction in vWF:Ag levels by addition of dipyridamole to Aspirin on day 14<br>- No reduction in vWF:pp levels in any group

Abbreviations: CE, cardioembolic; FVIII:C, factor VIII concentration; HR, hazard ratio; IS, ischaemic stroke; NIHSS, National Institute of Health Stroke Scale; OR, odds ratio; SD, standard deviation; TIA, transient ischaemic attack; vWF, von Willebrand factor; vWF:Ag, antigen; vWF:pp, propeptide.

Note: We searched Pubmed up to January 1, 2018, using the keywords 'vWF', 'ischaemic stroke', 'risk' and 'outcome' and reviewed previous studies investigating the association between vWF:Ag and pp levels as well as vWF activity measurements and the risk of and outcome in ischaemic stroke. We identified five observational studies, which are summarized in this table.

Supplementary Table S3 Animal studies of investigational vWF inhibitors

Animal studies					
**Name**	**Type**	**Target**	**Model**	**Outcome**	**PMID Reference**
Ajvw-2 Fa	Monoclonal antibody	GPIbα-vWF	Dog	Coronary artery thrombosis	11297756
82D6A3	Monoclonal antibody	Fibrillar collagen type I/III - vWF	Baboon	Arterial thrombosis	11986216
GPG-290	Recombinant chimeric protein linked to human Fc	GPIbα-vWF	Dog	Coronary thrombosis	17721623
h6B4-Fab	Monoclonal antibody	GPIbα-vWF	Baboon	Arterial thrombosis	18841291
ARC15105	Aptamer	GPIbα-vWF	Monkey	Pharmacokinetics and pharmacodynamics	22282355
MHCSZ-123	Monoclonal antibody	Collagen type III -vWF	Monkey	Arterial thrombosis	28526067
SZ-123 (MHCSZ-123)	Monoclonal antibody	Fibrillar collagen type III - vWF	Rhesus Monkey	arterial thrombosis	23295157<br>28526067

Abbreviations: GP, glycoprotein; vWF, von Willebrand factor.
Note: Including only those which have not yet been investigated in humans.
